# Elimination of Hepatitis C Virus from Hepatocytes by a Selective Activation of Therapeutic Molecules

**DOI:** 10.1371/journal.pone.0015967

**Published:** 2011-01-06

**Authors:** Xiaoyu Wen, Takayuki Abe, Hiroshi Kukihara, Shuhei Taguwa, Yoshio Mori, Hideki Tani, Nobuyuki Kato, Tetsuro Suzuki, Masashi Tatsumi, Kohji Moriishi, Yoshiharu Matsuura

**Affiliations:** 1 Department of Molecular Virology, Research Institute for Microbial Diseases, Osaka University, Osaka, Japan; 2 Department of Tumor Virology, Okayama University Graduate School of Medicine, Dentistry, and Pharmaceutical Sciences, Okayama, Japan; 3 Department of Infectious Diseases, Hamamatsu University School of Medicine, Hamamatsu, Japan; 4 AIDS Research Center, National Institute of Infectious Diseases, Tokyo, Japan; University of Cambridge, United Kingdom

## Abstract

To eliminate hepatitis C virus (HCV) from infected hepatocytes, we generated two therapeutic molecules specifically activated in cells infected with HCV. A dominant active mutant of interferon (IFN) regulatory factor 7 (IRF7) and a negative regulator of HCV replication, VAP-C (Vesicle-associated membrane protein-associated protein subtype C), were fused with the C-terminal region of IPS-1 (IFNβ promoter stimulator-1), which includes an HCV protease cleavage site that was modified to be localized on the ER membrane, and designated cIRF7 and cVAP-C, respectively. In cells expressing the HCV protease, cIRF7 was cleaved and the processed fragment was migrated into the nucleus, where it activated various IFN promoters, including promoters of IFNα6, IFNβ, and IFN stimulated response element. Activation of the IFN promoters and suppression of viral RNA replication were observed in the HCV replicon cells and in cells infected with the JFH1 strain of HCV (HCVcc) by expression of cIRF7. Suppression of viral RNA replication was observed even in the IFN-resistant replicon cells by the expression of cIRF7. Expression of the cVAP-C also resulted in suppression of HCV replication in both the replicon and HCVcc infected cells. These results suggest that delivery of the therapeutic molecules into the liver of hepatitis C patients, followed by selective activation of the molecules in HCV-infected hepatocytes, is a feasible method for eliminating HCV.

## Introduction

Hepatitis C virus (HCV) is a major cause of chronic liver diseases. A high risk of chronicity is the major concern of HCV infection, since chronic HCV infection often leads to liver cirrhosis and hepatocellular carcinoma [1], [2]. Although the proportion of patients achieving a sustained virological response (SVR) has been increased by the recent used of combination therapy with pegylated-interferon-α (PEG-IFNα) and ribavirin (RBV), half of patients still exhibit no response to this therapy, suggesting that the IFN signaling pathway is modulated by HCV infection. In addition, various side effects have been reported in more than 20% of patients treated with this combination therapy [3].

HCV belongs to the family *Flaviviridae* and possesses a single positive-stranded RNA genome that encodes a single polyprotein composed of about 3,000 amino acids. The HCV polyprotein is processed into 10 viral proteins by host and viral proteases. Viral structural proteins, including the capsid protein and two envelope proteins, are located in the N-terminal one third of the polyprotein, followed by nonstructural proteins. The NS2 protease cleaves its own carboxyl terminus and NS3 cleaves the downstream positions to produce NS4A, NS4B, NS5A and NS5B. Although laboratory strains of HCV propagating in cell culture (HCVcc) have been established based on the full-length genome of the genotype 2a JFH1 strain [4], establishment of a robust cell culture system capable of propagating serum-derived HCV from hepatitis C patients has not yet been achieved.

Type I IFN exhibits potent antiviral effects through the regulation of hundreds of IFN-stimulated genes (ISGs) which encode proteins involved in the establishment of antiviral state in cells [5]. IFNs induce transcription of ISGs through activation of the Jak-STAT pathway [6]. Binding of type I IFN to the IFN receptor induces phosphorylation of the receptor-associated tyrosine kinases, Jak1 and Tyk2, and then these kinases activate STAT1 and STAT2. The phosphorylated STATs migrate into the nucleus and activate ISG promoters through binding to the specific responsible elements. HCV infection has been suggested to impair the IFN production through multiple pathways. The IFN-induced Jak-STAT signaling is inhibited in cells and transgenic mice expressing HCV proteins and in the liver biopsy samples of chronic hepatitis C patients [7]–[9].

Induction of type I IFN upon infection with pathogens is crucial for innate immunity, and it is mediated by the activation of pattern-recognition receptors, including Toll-like receptors (TLRs) and cytosolic receptors, such as RIG-I and MDA5 [10]–[12]. The induction of type I IFN is primarily controlled at the gene transcriptional level, wherein a family of transcription factors known as IFN regulatory factors (IRFs) play a pivotal role. IRF3 and IRF7 are known to be essential for the RIG-I-, MDA5-, and TLR-mediated type I IFN production pathways. IRF3 is induced primarily by a response to initiate IFNβ production, whereas IRF7 is induced by IFNβ and participates in the late phase for IFNβ induction [13]. All TLRs, except for TLR3, activate the MyD88-dependent pathway, whereas TLR3 and TLR4 activate the TRIF-dependent pathway. HCV NS3/4A protease has been shown to impair the production of IFNβ as well as the subsequent IFN-inducible genes through the inactivation of the adaptor molecules involved in the TLR-dependent and -independent signaling pathways [14]–[18]. On the other hand, Vilasco *et al.* suggested that impairment of IKKi - which, along with TBK1, is one of the important factors participating in IRF3 phosphorylation and activation - in the HCV replicon cells plays at least a partial role in the restoration of type I IFN signaling pathways [19]. In addition, IRF7 was shown to participate in the positive feedback of type I IFN signaling through the IFN receptor [13]. Therefore, we tried to examine the effect of exogenous expression of IRF7 under the assumption that IRF7 is a potent type I IFN inducer and capable of modulating the viral propagation in hepatocytes infected with HCV.

In this study, we generated two therapeutic molecules consisting of a dominant active mutant of IRF7 or VAP-C, a negative regulator of HCV replication [20], followed by the C-terminal region of IFN promoter stimulator 1 (IPS-1), including the cleavage site of the HCV NS3/4A protease, which was modified so that the cleavage site localized on the ER membrane [21]. The expression of the plasmids encoding these molecules in the HCV replicon and HCVcc-infected cells resulted in a substantial suppression of HCV propagation, suggesting the possibility that these or other similar molecules could be used therapeutically to eliminate HCV from hepatocytes infected with HCV.

## Results

### IRF7m, a dominant active mutant of IRF7, activates the IFN promoters in cells replicating HCV

**Figure 1:**
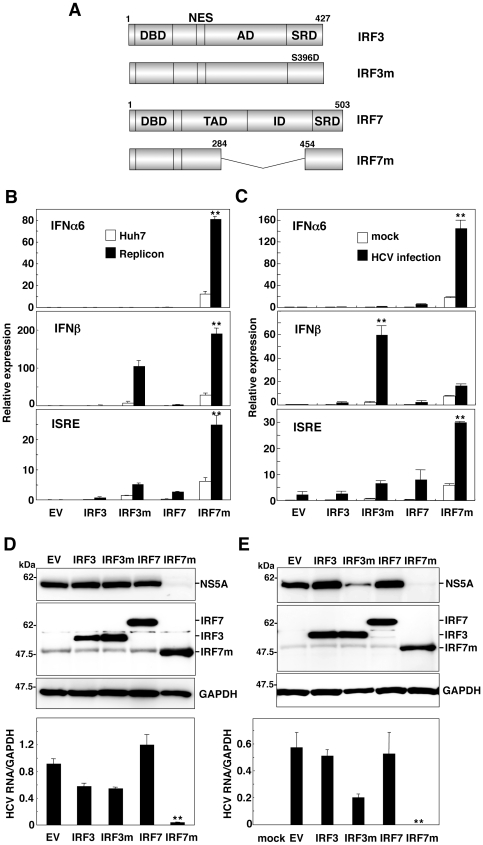
Dominant active mutant of IRF7 activates IFN promoters in cells replicating HCV. (A) Structures of IRF3, IRF7 and the dominant active mutants, IRF3m and IRF7m. The DNA-binding domain, nuclear export sequence, transactivation domain, association domain, inhibitory domain, and signal response domain are indicated as DBD, NES, TAD, AD, ID, and SRD, respectively. Huh7 cells and HCV replicon cells (1×10^5^ cells/well) (B), and Huh7OK1 cells (7.5×10^4^ cells/well) infected with HCVcc at an moi of 1 and incubated for 72 h (C) were transfected with 100 ng of plasmid encoding the luciferase gene under the control of the IFNα6, IFNβ, or ISRE promoter together with an empty vector (EV) or a plasmid encoding each of the IRF constructs. The relative luciferase activity of cell lysates was determined at 24 h post-transfection. HCV replicon cells (3×10^5^ cells/well) (D) and Huh7OK1 cells (1.5×10^5^ cells/well) infected with HCVcc at an moi of 1 and incubated for 72 h (E) were transfected with EV or a plasmid encoding each of the IRF constructs and the expressions of NS5A, IRFs, and GAPDH (upper panel) and synthesis of viral RNA (lower panel) at 72 h post-transfection were determined by immunoblotting and real-time PCR after standardization with GAPDH, respectively. The data shown in this figure are representative of three independent experiments. The error bars represent the standard deviations. Asterisks indicate significant differences (**P<0.01) versus the control cells or mock-infected cells.

Previous studies have shown that an IRF7 mutant, IRF7m, lacking the amino acid residues from 284 to 454, a region that includes the auto-inhibitory domain (from amino acid residue 305 to 467), and an IRF3 mutant, IRF3m, carrying the substitution of Ser^396^ to Asp in the carboxyl terminal region (Fig. 1A), induced a potent activation of type I IFN promoter in non-hepatic cell lines irrespective of viral infection [22]–[25]. We first examined the effect of the expression of the IRF dominant active mutants on the inhibition of HCV RNA replication through the production of type I IFN. HCV replicon cells and Huh7OK1 cells infected with HCVcc were transfected with the plasmids encoding either wild-type or dominant active mutant of IRF3 or IRF7 together with the reporter plasmids encoding a luciferase gene under the control of the promoters of IFNα6, IFNβ and ISRE, respectively. Among these examined constructs, we observed significant activation of the promoters of IFNα6 and ISRE in the replicon and HCVcc-infected cells compared with naïve and mock-infected cells upon expression of IRF7m, while we observed no activation of the IFNα6 promoter in cells expressing IRF3m (Figs. 1 B and 1C). Potent stimulation of the IFNβ promoter was observed in the replicon cells expressing IRF7m but not in cells infected with HCVcc. Next we examined the antiviral activity of the IRF constructs in both replicon (Fig. 1D) and HCVcc-infected cells (Fig. 1E). The expression of the plasmid encoding IRF7m resulted in potent suppression of viral protein and viral RNA syntheses in both cell types. Although expression of IRF3m induced a slight suppression of viral propagation in cells infected with HCVcc, expression of the IRF constructs except for IRF7m did not induce the significant suppression of viral replication and propagation. These results suggest the possibility of elimination of HCV through a specific induction of type I IFN by the expression of IRF7m in HCV-infected cells.

### cIRF7, a chimeric construct of IRF7m, specifically activates the IFN promoters in cells replicating HCV

**Figure 2:**
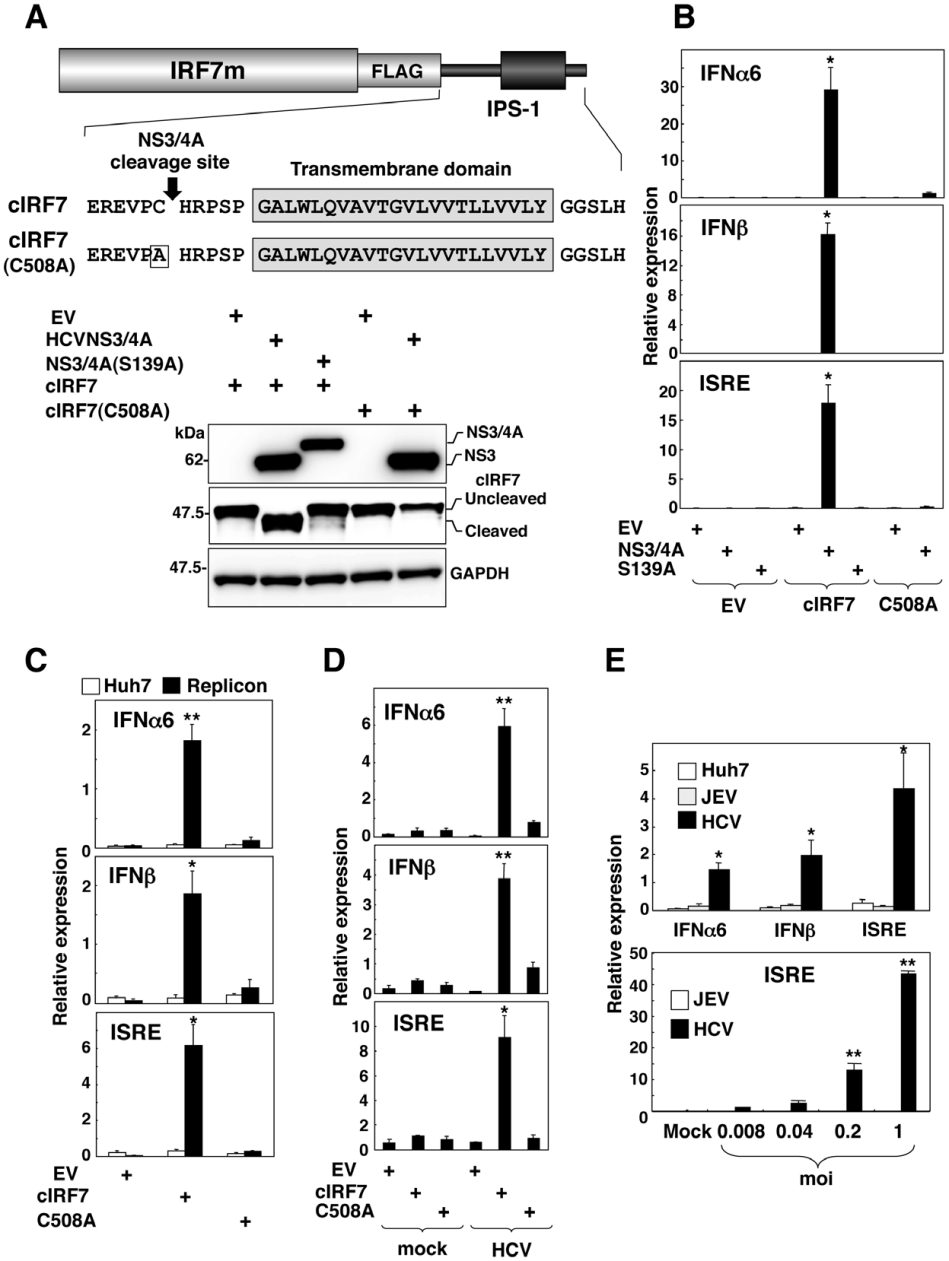
Construction of cIRF7 capable of activating the IFN promoters in cells replicating HCV. (A top) Schematic representation of the cIRF7 constructs. cIRF7 consists of IRF7m, FLAG-tag, and IPS-1 (503 to 540 amino acid residues) sequences containing a cleavage site by HCV NS3/4A protease, a transmembrane domain and a cytoplasmic region modified to localize on the ER. cIRF7(C508A) has a substitution of Cys508 to Ala which renders it resistant to the cleavage by the HCV protease. (A bottom) Immunoblot analyses of 293T cells transfected with a plasmid encoding either cIRF7 or cIRF7(C508A) together with either an empty vector (EV) or a plasmid encoding either FLAG-tagged HCVNS3/4A or FLAG-tagged HCVNS3/4A (S139A). (B) 293T cells (2×10^5^ cells/well) were transfected with a plasmid of EV, FLAG-tagged HCVNS3/4A or FLAG-tagged HCVNS3/4A(S139A) in combination with a plasmid of EV, cIRF7 or cIRF7 (C508A) together with 100 ng of the reporter plasmid encoding the luciferase gene under the control of the IFNα6, IFNβ or ISRE promoter, and luciferase activity was determined at 24 h post-transfection. (C) HCV replicon cells (1.5×10^5^ cells/well) and (D) Huh7OK1 cells (7.5×10^4^ cells/well) infected with HCVcc at an moi of 1 and incubated for 72 h were transfected with 100 ng of each of the reporter plasmids together with plasmid of EV, cIRF7 or cIRF7(C508A) and luciferase activity was determined at 24 h post-transfection. (E) Huh7 cells, HCV subgenomic replicon cells, and JEV subgenomic replicon cells (1×10^5^ cells/well) (top) and Huh7OK1 cells (7.5×10^4^ cells/well) infected with JEV and HCV (bottom) at an moi of 0.008, 0.04, 0.2, and 1 and incubated for 24 h and 72 h, respectively, were transfected with 100 ng of each of the reporter plasmids together with cIRF7 and the luciferase activity was determined at 24 h post-transfection. The data shown in this figure are representative of three independent experiments. The error bars represent the standard deviations. Asterisks indicate significant differences (*P<0.05, **P<0.01) versus the control cells or mock-infected cells.

**Figure 3:**
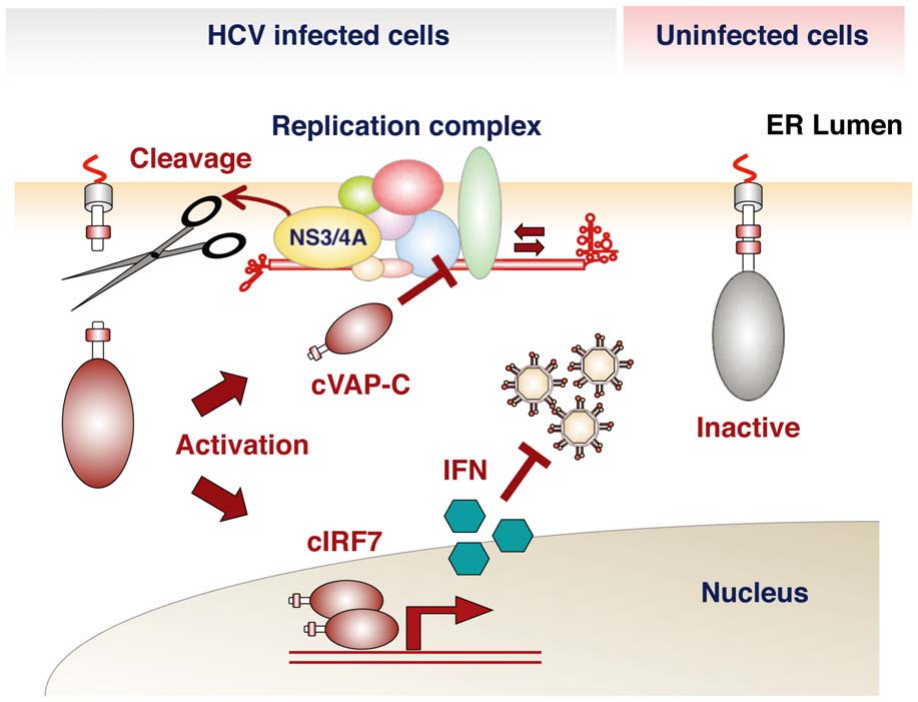
Scheme of activation of the therapeutic molecule in cells infected with HCV. The chimeric molecules are cleaved by HCV NS3/4A protease and the released fragments inhibit propagation of HCV through induction of IFN after translocation into the nucleus (cIRF7) or disruption of the replication complex (cVAP-C), whereas the molecule is stably anchored in the ER within uninfected cells.

To induce IFNs in cells infected with HCV but not in uninfected cells through a selective activation of IRF7m, we constructed a chimeric IRF7 (cIRF7) consisting of the IRF7m fused with FLAG-tag and the C-terminal amino acid residues from 503 to 540 of IPS-1 modified to be localized on ER (Fig. 2A upper) [21]. HCV NS3/4A protease cleaves the carboxyl site of Cys^508^ in the C-terminal domain of IPS-1. Although cIRF7 is anchored in the ER and exhibits no activation in uninfected cells, cIRF7 would be cleaved by the NS3/4A protease in cells infected with HCV and the released N-terminal fragment would migrate into the nucleus and activate various IFN promoters (Fig. 3). Immunoblot analyses revealed that cIRF7 was cleaved in 293T cells expressing HCV NS3/4A protease of a wild type but not in those expressing the mutant protease NS3/4A(S139A), and a mutant cIRF7(C508A) which has a substitution of Cys^508^ to Ala, exhibited resistance to the cleavage by the HCV protease (Fig. 2A bottom). To assess a specific activation of the IFN promoters after cleavage of the cIRF7 by HCV NS3/4A, 293T cells expressing FLAG-tagged HCV proteases were transfected with the plasmids encoding the luciferase gene under the control of the promoter of IFNα6, IFNβ or ISRE together with the plasmid encoding either cIRF7 or cIRF7(C508A). Expression of cIRF7 but not of cIRF7(C508A) induced the activation of the IFNα6, IFNβ and ISRE promoters in cells expressing HCV NS3/4A protease but not in those expressing the mutant protease NS3/4A(S139A) (Fig. 2B). Next we examined the activation of the IFN promoters associated with the expression of the plasmid encoding cIRF7 in the replicon and HCVcc-infected cells. Expression of cIRF7 but not of cIRF7(C508A) induced the activation of the IFN promoters in both cell types (Figs. 2C and 2D). On the other hand, these promoters were not activated by the expression of cIRF7 in the replicon cells harboring subgenomic RNA of Japanese encephalitis virus (JEV) and Huh7 cells infected with JEV (Fig. 2E). These results suggest that the cIRF7 expression is a feasible method for specifically activating the IFN promoters in cells infected with HCV.

### Specificity of activation of the IFN promoters by the expression of cIRF7

**Figure 4:**
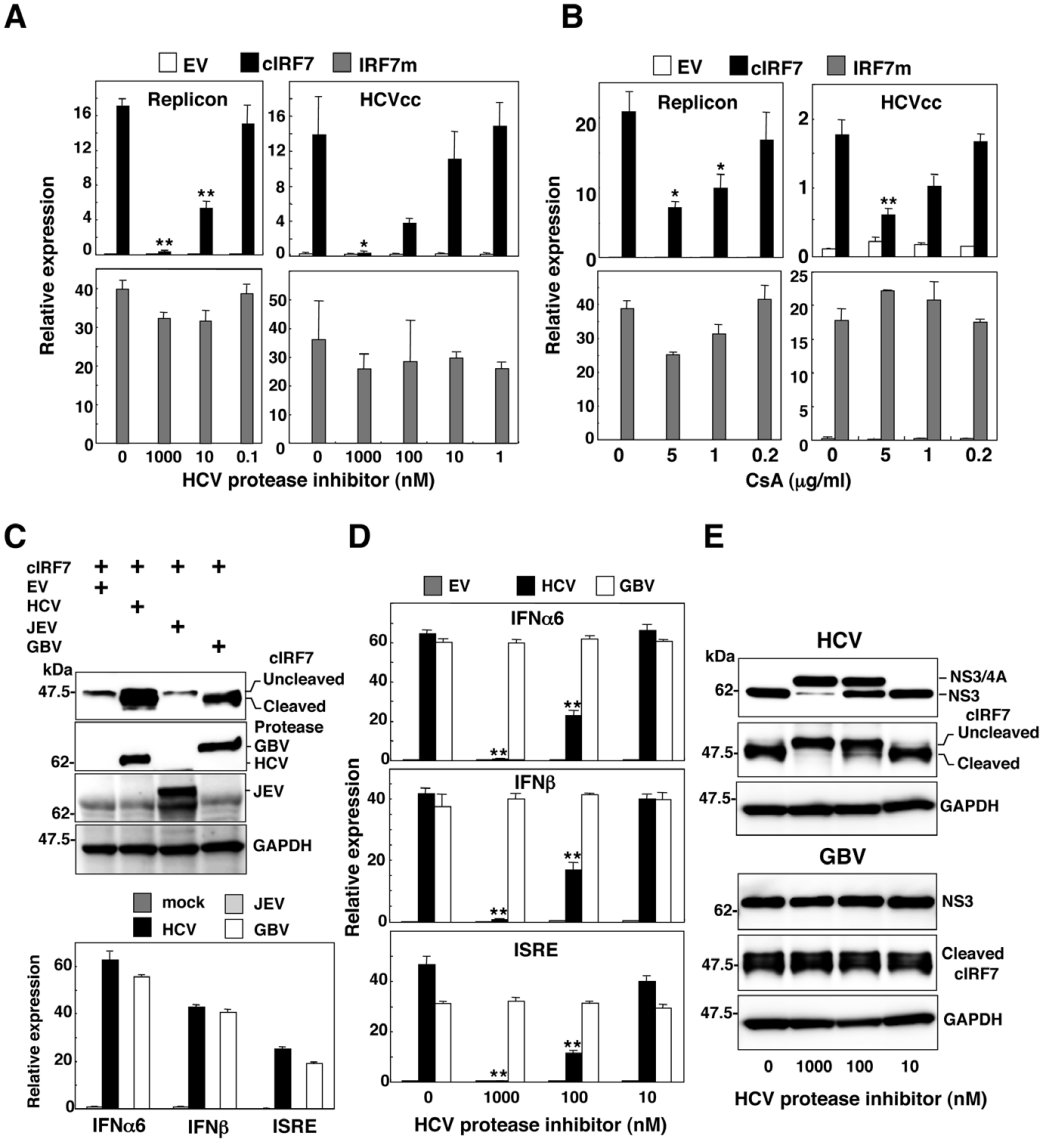
Specificity of activation of the IFN promoters by the expression of cIRF7. (A) HCV replicon cells (1.5×10^5^ cells/well) or Huh7OK1 cells (7.5×10^4^ cells/well) infected with HCVcc at an moi of 1 and incubated for 72 h were treated with various concentrations of HCV protease inhibitor (A) or cyclosporine A (CsA) (B), transfected with an empty vector (EV) (white bars) or plasmids encoding cIRF7 (black bars) or IRF7m (gray bars) together with 100 ng of a reporter plasmid encoding the luciferase gene under the control of the ISRE promoter, and luciferase activity was determined at 24 h post-transfection. (C top) A plasmid encoding cIRF7 was co-transfected with a plasmid encoding either FLAG-tagged HCVNS3/4A, FLAG-tagged GBVNS3/4A, or HA-tagged JEVNS2b/3 into 293T cells, and the expressions of cIRF7, viral proteases and GAPDH were determined by immunoblotting. (C bottom) 293T cells (2×10^5^ cells/well) transfected with a plasmid encoding either EV (dark gray bars), FLAG-tagged HCVNS3/4A (black bars), FLAG-tagged GBVNS3/4A (white bars), or HA-tagged JEVNS2b/3 (gray bars) together with 100 ng of the plasmid encoding the luciferase gene under the control of the promoter of either IFNα6, IFNβ or ISRE, and luciferase activity was determined at 24 h post-transfection. (D) 293T cells (2×10^5^ cells/well) were transfected with 100 ng of the reporter plasmids together with plasmids encoding EV (gray bars), FLAG-tagged HCVNS3/4A (black bars) or FLAG-tagged GBVNS3/4A (white bars) in the presence or absence of the HCV protease inhibitor, and luciferase activity was determined at 24 h post-transfection. (E) cIRF7 was co-expressed with FLAG-tagged HCVNS3/4A or FLAG-tagged GBVNS3/4A in 293T cells in the presence or absence of the HCV protease inhibitor, and the expressions of cIRF7, viral proteases and GAPDH were determined by immunoblotting. The data shown in this figure are representative of three independent experiments. The error bars represent the standard deviations. Asterisks indicate significant differences (*P<0.05, **P<0.01) versus the control cells or mock-infected cells.

To further examine the specificity of the activation of the IFN promoters by the expression of cIRF7 in cells replicating HCV, a plasmid encoding either cIRF7 or IRF7m was co-transfected with that encoding the luciferase gene under the ISRE promoter into the HCV replicon or HCVcc-infected cells and cultured in the presence or absence of inhibitors for HCV replication. Treatment with an HCV protease inhibitor (BILN2061) or cyclosporine A (CsA) inhibited the activation of the ISRE promoter by the expression of cIRF7 in the HCV replicon and HCVcc-infected cells in a dose-dependent manner, in contrast to the resistance to the treatments in cells expressing the IRF7m (Fig. 4A and Fig. 4B). Recently, it was shown that an NS3/4A protease of GB virus B (GBV-B), which is the virus genetically related most closely to HCV, also impairs the dsRNA-induced IFN production through a cleavage of IPS-1[26]. Therefore, to assess the possibility of activation of cIRF7 by other flaviviral proteases, cleavage of cIRF7 and activation of the IFN promoters were evaluated in 293T cells expressing the viral proteases of HCV, GBV and JEV. Immunoblot analyses revealed that cIRF7 was processed by the viral proteases of HCV and GBV but not by that of JEV and the activation of the IFN promoters was well correlated with the cleavability of the cIRF7 (Fig. 4C). Although the GBV protease exhibited an efficient activation of cIRF7 comparable to HCV protease, processing of cIRF7 and activation of the IFN promoters by the GBV protease was not inhibited by the pretreatment with the HCV protease inhibitor (Figs. 4D and 4E). These results indicate that cIRF7 is capable of activating the IFN promoters through a specific cleavage by the protease in cells infected with HCV.

### Nuclear localization of cIRF7 in cells expressing HCV protease

**Figure 5:**
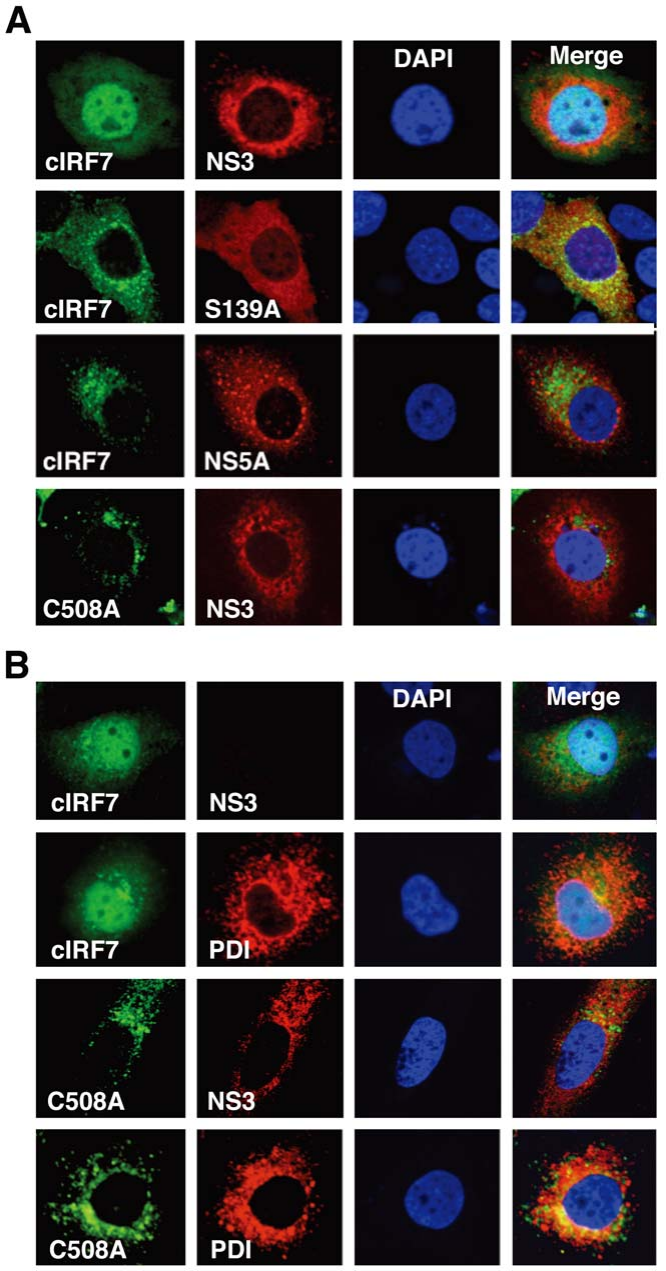
Activation of cIRF7 in cells expressing HCV protease. (A) Huh7OK1 cells (5×10^4^ cells/well) were co-transfected with plasmids encoding either EGFP-cIRF7 or EGFP-cIRF7(C508A) and plasmids encoding either HCVNS3/4A, HCVNS3/4A(S139A) or NS5A, harvested at 24 h post-transfection, fixed with 4% paraformaldehyde in PBS, and permeabilized with 0.25% saponin. HCV NS3 and NS5A were stained with the appropriate antibodies, followed by staining with AF594-conjugated second antibodies. (B) HCV replicon cells (5×10^4^ cells/well) were transfected with plasmids encoding either EGFP-cIRF7 or EGFP-cIRF7(C508A), and endogenous expression of HCV NS3 and an ER marker, PDI, was detected in cells treated and stained with the appropriate antibodies as described above. Subcellular localization of cIRF7s, HCV proteins and PDI was determined by confocal microscopy after staining of nuclei by DAPI. The data shown in this figure are representative of three independent experiments.

From these results, it was suggested that cIRF7 is cleaved by the HCV protease and the processed fragment migrates into the nucleus and activates IFN promoters (Fig. 3). To confirm the nuclear localization of the cleaved cIRF7, we constructed an EGFP-cIRF7 and determined its subcellular localization in cells expressing the HCV protease and in the HCV replicon cells by confocal microscopy. Nuclear accumulation of the cIRF7 was observed in cells expressing EGFP-cIRF7 together with NS3/4A, but not in those with NS3/4A(S139A) or NS5A and also not in cells co-expressing EGFP-cIRF7(C508A) and NS3/4A (Fig. 5A). Furthermore, expression of EGFP-cIRF7 but not of EGFP-cIRF7(C508A) induced a nuclear accumulation of cIRF7 in the HCV replicon cells, and nuclear localization of the cIRF7 abrogated the expression of viral antigen (NS3), in contrast to the co-localization of EGFP-cIRF7(C508A) and the ER marker PDI, which had no discernible antiviral effect (Fig. 5B). These results suggest that cIRF7 is capable of suppressing HCV replication through an HCV protease-dependent cleavage, migration into the nucleus and activation of the IFN promoters.

### Suppression of HCV replication by the expression of cIRF7

**Figure 6:**
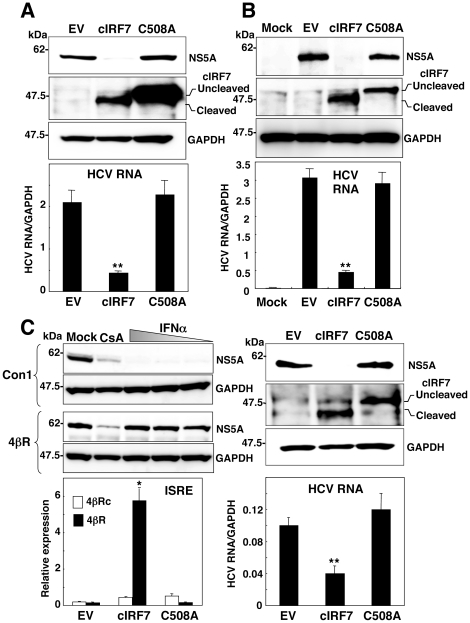
Suppression of HCV replication by the expression of cIRF7. (A) HCV replicon cells (3×10^5^ cells/well) and (B) Huh7OK1 cells (1.5×10^5^ cells/well) infected with HCVcc at an moi of 1 and incubated for 72 h were transfected with a plasmid encoding either empty vector (EV), cIRF7 or cIRF7(C508A), and the expression of NS5A, cIRF7s and GAPDH (upper panels) and synthesis of viral RNA (lower panels) were determined at 72 h post-transfection by immunoblotting and real-time PCR, respectively. (C upper left) HCV Con1 replicon cells and 4βR replicon cells exhibiting an IFN-resistant phenotype (1.5×10^5^ cells/well) were treated with the CsA (5 µg/ml) or 10^4^, 10^3^, and 10^2^ units/ml of recombinant human IFNα and the expressions of NS5A and GAPDH were determined by immunoblotting. The 4βR replicon cells (3×10^5^ cells/well) were transfected with EV or plasmid encoding either cIRF7 or cIRF7(C508A), and the expressions of NS5A, cIRF7s and GAPDH (C upper right) and synthesis of viral RNA (C lower right) were determined at 72 h post-transfection by immunoblotting and real-time PCR, respectively. The 4βR cells and their cured cells (4βRc) with the HCV genome eliminated (1×10^5^ cells/well) were transfected with EV or plasmid encoding either cIRF7 or cIRF7(C508A) together with 100 ng of plasmid encoding the luciferase gene under the control of the ISRE promoter, and luciferase activity was determined at 24 h post-transfection (C lower left). The data shown in this figure are representative of three independent experiments. The error bars represent the standard deviations. Asterisks indicate significant differences (*P<0.05, **P<0.01) versus the control cells or mock-infected cells.

To examine the inhibitory effect of the expression of cIRF7 on HCV replication, a plasmid encoding either cIRF7 or cIRF7(C508A) was transfected into the HCV replicon and HCVcc-infected cells, and HCV replication was evaluated by immunoblotting and real-time PCR. The expression of cIRF7 but not of cIRF7(C508A) resulted in cleavage by the HCV protease, and a clear reduction of viral protein and RNA syntheses in both replicon and HCVcc-infected cells (Figs. 6A and 6B). In addition, we examined the effect of cIRF7 on the replication of HCV in the 4βR replicon cells [27], [28], which have been shown to exhibit more resistant to the IFNα treatment than Con1 replicon cells (Fig. 6C upper left). Expression of the cIRF7 in the 4βR replicon cells but not in those cured HCV RNA (4βRc cells) induced an activation of the ISRE promoter (Fig. 6C lower left). Expression of cIRF7 but not of cIRF7(C508A) also resulted in processing by the HCV protease and suppression of viral protein and RNA syntheses in the 4βR replicon cells (Fig. 6C right panels).

### Suppression of HCV replication by the expression of cVAP-C

**Figure 7:**
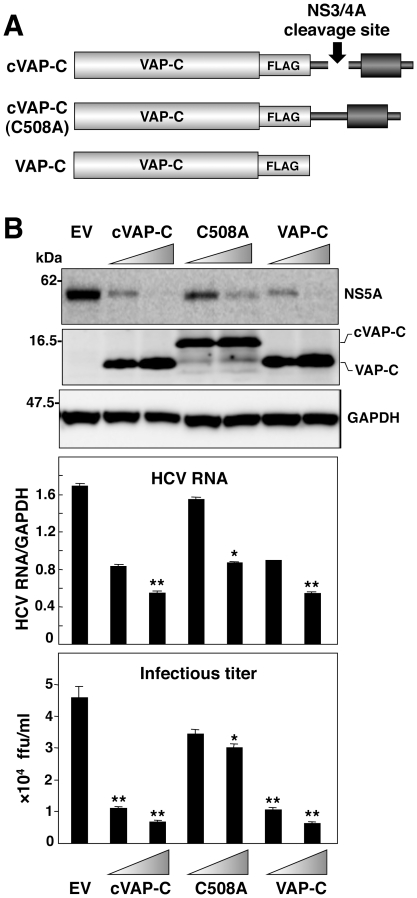
Suppression of HCV replication by the expression of cVAP-C. (A) Schematic representation of cVAP-C, cVAP-C(C508A) and VAP-C. Like cIRF7, cVAP-C is composed of the sequences of VAP-C, FLAG-tag, and the C-terminus domain of IPS-1. (B) Huh7OK1 cells (1.5×10^5^ cells/well) infected with HCVcc at an moi of 1 and incubated for 72 h were transfected with EV, or plasmid encoding either cIRF7 or cIRF7(C508A), and the expressions of NS5A, VAP-Cs and GAPDH (top panel), synthesis of viral RNA (middle panel) and infectious titers in the culture supernatants were determined at 72 h post-transfection by immunoblotting, real-time PCR, and focus forming assay, respectively. The data shown in this figure are representative of three independent experiments. The error bars represent the standard deviations. Asterisks indicate significant differences (*P<0.05, **P<0.01) versus the control cells or mock-infected cells.

Human vesicle-associated membrane protein-associated protein subtype A (VAP-A) and B (VAP-B) are known to be involved in the regulation of membrane trafficking, lipid transport and metabolism, and the unfolded protein response [29]. VAP-A and VAP-B have been shown to be involved in the replication of HCV, and we have shown recently that human VAP-C, a splicing variant of VAP-B, negatively regulates HCV replication by interfering with the interaction of VAP-A and VAP-B with HCV NS5B [20]. We next examined the possibility of using a selective activation of VAP-C to suppress HCV replication in cells infected with HCVcc. We generated expression plasmids encoding a chimeric VAP-C fused with the IPS-1 sequence (cVAP-C), a cVAP-C(C508A) which is made resistant to the HCV protease by a substitution in the cleavage site similar to the substitutions made in cIRF7(C508A), or VAP-C (Fig. 7A). The cVAP-C was cleaved in cells infected with HCVcc, and expression of cVAP-C and VAP-C suppressed expression of NS5A, in contrast to the weak reduction of NS5A in the infected cells expressing cVAP-C(C508A), probably due to a slight cleavage of cVAP-C(C508A) (Fig. 7B, top). Furthermore, the production of viral RNA and infectious particles in the culture supernatants of cells infected with HCVcc was also impaired by the expression of cVAP-C and VAP-C, but not of cVAP-C(C508A) in a dose-dependent manner (Fig. 7B, middle and bottom). Collectively, these results suggest that delivery of the therapeutic molecules into liver of hepatitis C patients, followed by selective activation of the molecules in HCV-infected hepatocytes, is a feasible method for eliminating HCV.

## Discussion

An effective prophylactic vaccine against HCV has not been developed yet. Although combination therapy consisting of PEG-IFNα and RBV has been introduced for the treatment of hepatitis C patients, and 50% of individuals infected with genotype 1 achieved a SVR, this treatment is sometimes associated with serious side effects, including depression and anemia [3]. Therefore, new anti-HCV drugs targeted to HCV protease and polymerase and capable of optimizing therapy are currently in the early stages of the development [30], [31]. However, it is difficult to achieve a complete removal of viruses by antiviral drugs targeted to the viral enzymes from patients persistently infected with RNA viruses that exhibit a quasispecies nature, such as human immunodeficiency virus (HIV) and HCV. Viral quasispecies are not a simple collection of diverse mutants but a group of interactive variants capable of adapting to new environments [32]. Furthermore, introduction of antiviral drugs may induce an emergence of drug-resistant breakthrough viruses as seen in the case of HIV infection. Therefore, a novel therapeutic approach for hepatitis C patients in addition to the current chemotherapies is required to overcome serious adverse effects and improve the ratio of patients achieving SVR.

In this study, we have generated two therapeutic molecules, cIRF7 and cVAP-C, which are selectively activated in cells replicating HCV. To tightly regulate activation of the molecules in HCV- infected cells, we employed the C-terminal amino acid sequence of human IPS-1, which has been identified as an adaptor molecule involved in the RIG-like receptor (RLR) signaling pathways. It has been demonstrated that HCV NS3/4A protease efficiently cleaves the upstream position of the transmembrane region of IPS-1 on the mitochondrial outer membrane and disrupts the IFN signaling pathway [15]–[18]. Furthermore, to avoid induction of mitochondrial dysfunction and cell death due to the expression of the therapeutic molecules on the mitochondria, we replaced three arginine residues among the C-terminal five residues of IPS-1 with non-charged amino acid glycine residues ( RRRLH to GGGLH) so that these three residues would be localized on the ER membrane [21]. HCV is suggested to replicate on the ER membrane, and therefore subcellular localization and distance of the cleavage site of the substrates from the membrane could be crucial for an efficient processing. The tightly regulated activation of the therapeutic molecules in cells replicating HCV observed in this study might be largely attributable to the ER localization of the therapeutic molecules.

Irrespective of IFN sensitivity, the expression of cIRF7 in the HCV replicon cells induced the activation of type I IFN promoter and inhibited the viral RNA replication, suggesting the possibility that cIRF7 could be used for the treatment of hepatitis C patients who are infected with HCV resistant to IFNα therapy. The expression of IRF3m in cells infected with HCVcc induced a higher antiviral response than that in the Con1 replicon cells in spite of the comparable transcription of IFNβ mRNA between the two cell types Fig. 1), suggesting that differences among HCV genotypes might be caused to the difference to the sensitivity of IFNβ. To assess the real efficacy of cIRF7 for suppression of HCV replication, we must await the establishment of robust cell culture systems capable of propagating various genotypes of HCV derived from the sera of hepatitis C patients.

It has been shown previously that HCV interferes with the induction of type I IFN through the cleavage of IPS-1 by NS3/4A protease [15]–[18], the interaction of NS5A with MyD88, a major adaptor molecule of TLRs [33], and the intervention of the IFNα-activated Jak-STAT signaling pathway by HCV proteins [7]–[9]. After cleavage by the HCV protease, the processed cIRF7 migrates into the nucleus and activates various IFN promoters, and it may participate in regulation of the expression of hundreds of ISGs, suggesting that cIRF7 is capable of inducing an antiviral response through the Jak-STAT-independent pathway. Although it has been reported previously that the basal expression of IRF7 and the IRF7-induced activation of the IFNα promoter are impaired in the HCV replicon cells [34], in this study we have shown that cIRF7 is activated in cells infected with HCVcc and capable of inducing type I IFN. Collectively, these results suggest that cIRF7 is capable of eliminating HCV that persistently infects human hepatocytes through an induction of sufficient amounts of type I IFN.

It is well known that patients achieving a rapid viral clearance by the treatment with PEG-IFNα showed a significant up-regulation of ISG, whereas a high level expression of ISG is observed in nonresponsive patients before IFN therapy, probably due to a rapid induction of negative regulators for the IFN signaling pathway, such as the suppressor of cytokine signaling proteins [35], [36]. These results suggest that chronic hepatitis C patients with a pre-activated IFN signaling pathway respond poorly to IFN therapy. In this study we also demonstrated that activation of various IFN promoters by the expression of the dominant active mutants of IRFs was more accentuated in cells replicating HCV rather than naïve cells, probably due to an undetectable expression of ISG in cells replicating HCV RNA as described previously [37]. However, the precise mechanisms underlying the enhancement of IFN activity by the expression of a dominant active mutant of IRFs in cells replicating HCV remain unknown. Fillipowicz *et al.* suggested the possibility of recovery of the sensitivity to IFN therapy by the restoration of the endogenous IFN system to a “naïve” state through a blockage of the IFN response in nonresponders before treatment [36]. However, modulation of ISG expression before IFN therapy may induce a flare of HCV propagation in the liver of chronic hepatitis C patients. Therefore, it might be interesting to examine whether an effectiveness of cIRF7 are sustained in a state of occurring a negative regulator for IFN signaling pathway and preactivated IFN signaling pathway in cells replicating HCV.

VAP-A and VAP-B are suggested to be involved in the construction of the HCV replication complex consisting of viral proteins and host cellular lipid components, and that VAP-C interrupts the VAP-A and VAP-B functions and negatively regulates the HCV propagation and not expressed in human hepatocytes probably involves in the determination of tissue tropism of HCV [20]. Although further studies will be needed to elucidate the effectiveness of the molecules *in vivo* experiment using drug delivery systems including viral and non-viral vectors in more detail, therapeutic molecules consisting of host factors involved in IFN induction such as IRF7 and in the suppression of HCV replication such as VAP-C fused with the IPS-1 sequences specifically cleaved by the HCV protease might be a promising approach capable of eliminating HCV without induction of severe cellular toxicity.

## Materials and Methods

### Cells and viruses

Vero and 293T cell lines were purchased from American Type Culture Collection (Manassas, VA). Huh7 cell line was kindly provided by Ralf Bartenschlager. Huh7OK1 cell line was previously established from interferone-treated Huh7 cells including HCV replicon and exhibited high susceptibility to HCVcc propagation [38]. These cell lines were maintained in Dulbecco's modified Eagle's medium (DMEM) (Sigma, St. Louis, MO) supplemented with 10% fetal calf serum (FCS). Huh-9–13 cells harboring an HCV subgenomic RNA replicon of genotype 1b [39] were cultured in DMEM supplemented with 10% FCS, 1 mg/ml G418 and nonessential amino acids. The infectious RNA of the JFH1 strain was introduced into Huh7OK1 cells and the infectious titers were expressed as focus-forming units (FFU) [4]. Huh7 cells harboring a JEV subgenomic RNA replicon (Nakayama strain) were cultured in DMEM supplemented with 10% FCS and 1 µg/ml puromycin. Preparation of the HCV subgenomic replicon cells 4βR exhibiting an IFN-resistant phenotype and their cured cells 4βRc were described previously [27], [28]. All cells were cultured at 37°C in a humidified atmosphere with 5% CO_2_.

### Plasmids and reagents

The cDNA fragments encoding IRF3 and IRF7 were amplified by PCR from a total RNA from THP-1 cells and cloned into pcDNA3.1-C-myc-His (Invitrogen, Carlsbad, CA). The mutants carrying a deletion in the auto-inhibitory domain (from amino acid residue 284 to 454) of IRF7 and the substitution of Ser^396^ with phosphomimetic Asp located in the carboxyl terminus of IRF3 were generated by the method of splicing by overlap extension and cloning into pcDNA3.1myc-His and designated as IRF7m and IRF3m, respectively. N-terminally FLAG-tagged wild-type NS3/4A protease and its mutant substituted with Ser^139^ to replaced with Ala (S139A) were prepared as described previously [33]. The cDNA fragment encoding a JEV protease was amplified from a total RNA of Vero cells infected with JEV (AT31 strain) and cloned into pcDNA3.1Flag/HA [40], The cDNA fragment encoding a GBV-B protease was amplified from pGBB (kindly provided by Dr. H. Akari) [41] by PCR and cloned into pcDNA3.1Flag/HA. The chimeric IRF7 (cIRF7) composed of the IRF7m fused with FLAG-tag and the C-terminus of human IPS-1 (from amino acid residues 503 to 540 amino acid residues) containing a cleavage site of HCV NS3/4A, transmembrane domain and the ER retention signal [21] (Fig. 2A) was cloned into pcDNA3.1-c-myc-His. A cIRF7 mutant, C508A, was generated to be resistant to HCV NS3/4A protease by substitution of Cys^508^ of cIRF7 to Ala. The reporter constructs of IFNα6, IFNβ, and ISRE were kindly provided by Drs. T. Kawai and S. Akira. All PCR products were confirmed by sequencing by an ABI PRISM 310 genetic analyzer (Applied Biosystems, Tokyo, Japan). The HCV NS3/4A protease inhibitor, BILN2061 was purchased from Acme Bioscience (Belmont, CA). Human recombinant IFNα and cyclosporine A (CsA) were purchased from PBL Biomedical Laboratories (New Brunswick, NJ) and Wako Pure Chemical Industries (Osaka, Japan), respectively.

### Reporter assay

Huh7 cells, HCV replicon cells, and Huh7OK1 cells infected with HCVcc were seeded onto 12-well plates at the concentration of 1.5×10^5^ cells/well and transfected with 100 ng of each of the plasmids encoding the luciferase gene under the control of the IFNα6, IFNβ and ISRE promoter together with the various constructs by using FuGene™6 (Roche Molecular Biochemicals, Mannheim, Germany). Luciferase activity was determined by the Dual-luciferase reporter assay system (Promega Inc., Madison, WI) and the *Renilla* luciferase reporter gene was simultaneously transfected as an internal control.

### Immunoblotting

HCV replicon cells and Huh7OK1 cells infected with HCVcc were transfected with the plasmids encoding each of the wild-type and the dominant active mutants of IRFs and harvested at 72 h post-transfection. Cells were washed three times with ice-cold phosphate-buffered saline (PBS), suspended in lysis buffer containing 20 mM Tris-HCl (pH 7.4), 135 mM NaCl, 1% Triton X-100, 10% glycerol and protease inhibitor cocktail tablets (Roche Molecular Biochemicals) and centrifuged at 14,000×g for 15 min at 4°C after incubation for 30 min at 4°C. Cell lysates were subjected to sodium dodecyl sulfate-12.5% polyacrylamide gel electrophoresis (SDS-PAGE) after boiling in sample buffer and transferred to polyvinylidene difluoride membranes (Millipore, Tokyo, Japan). The membranes were blocked with PBS containing 0.05% Tween 20 and 5% skim milk at room temperature for 1 h, incubated with mouse monoclonal anti-FLAG M2 (Sigma), anti-hemagglutinin (HA) 16B12 (HA.11; BabCO, Richmond, CA), anti-NS5A mouse monoclonal antibody (Austral Biologicals, San Ramon, CA), anti-GAPDH (Santa Cruz Biotechnology, Santa Cruz, CA), or anti-hexahistidine monoclonal antibody (Santa Cruz) at room temperature for 1 h, and then with horseradish peroxidase-conjugated anti-mouse IgG or anti-rabbit IgG antibody at room temperature for 1 h. The immune complexes were visualized with Super Signal West Femto substrate (Pierce, Rockford, IL) and detected by an LAS-3000 image analyzer system (Fujifilm, Tokyo, Japan).

### Quantitative reverse-transcription polymerase chain reaction (qRT-PCR)

A total RNA was prepared from HCV replicon cells and Huh7OK1 cells infected with HCVcc transfected with the plasmids encoding each of the IRF constructs using an RNeasy mini kit (QIAGEN, Valencia, CA) and first-strand cDNA was synthesized by using ReverTra Ace (TOYOBO, Osaka, Japan) and oligo (dT)_20_ primer. The expression of each cDNA was estimated by Platinum SYBR Green qPCR SuperMix UDG (Invitrogen) according to the manufacturer's protocol. Fluorescent signals were analyzed by an ABI PRISM 7000 (Applied Biosystems). The HCV and GAPDH genes were amplified using the primer pairs of 5′-GAGTGTCGTGCAGCCTCCA-3′ and 5′-CACTCGCAAGCACCCTATCA-3′, and 5′-ACCACAGTCCATGCCATCAC-3′ and 5′-TCCACCACCCTGTTGCTGTA-3′, respectively. The expression of each of mRNA was normalized with that of GAPDH.

### Subcellular localization of cIRF7 in HCV- replicating cells

Cells transfected with the plasmids were harvested at 24 h post transfection, washed twice with PBS, fixed with PBS containing 4% paraformaldehyde, and permeabilized by incubation with PBS containing 0.25% saponin for 10 min. Cells were incubated for 1 h at 4°C with 1µg/ml of anti-NS3 (251) mouse monoclonal antibody (Santa Cruz), anti-NS5A mouse monoclonal antibody (Austral Biologicals), or mouse monoclonal antibody to protein disulfide isomerase (PDI) (Affinity Bioreagents, Golden, CO) in PBS containing 10% FCS (PBSF), and then incubated at room temperature for 1 h with 0.5 µg/ml of Alexa Flour 594-conjugated anti-mouse IgG (Molecular Probes, Eugene, OR) after three time washes with PBSF. Cell nuclei were stained with 4′, 6-diamidino-2-phenylindole (DAPI). After an extensive wash with PBSF, the samples were examined with a Fluoview FV1000 laser scanning confocal microscope (OLYMPUS, Tokyo, Japan).

#### Statistical analysis

Results were expressed as the mean ± standard deviation. The significance of differences in the means was determined by Student's *t* test.
